# Role of the tumor microenvironment in shaping IDH-wildtype glioma plasticity, and potential therapeutic strategies

**DOI:** 10.20892/j.issn.2095-3941.2022.0363

**Published:** 2022-11-01

**Authors:** Lingxiang Wu, Ruichao Chai, Zheng Zhao, Qianghu Wang, Tao Jiang

**Affiliations:** 1 Department of Bioinformatics, Nanjing Medical University, Nanjing 211166, China; 2 Institute for Brain Tumors, Jiangsu Collaborative Innovation Center for Cancer Personalized Medicine, Nanjing Medical University, Nanjing 211166, China; 3 Department of Molecular Neuropathology, Beijing Neurosurgical Institute, Capital Medical University, Beijing 100070, China; 4 Chinese Glioma Genome Atlas Network (CGGA), Beijing 100070, China; 5 Department of Neurosurgery, Beijing Tiantan Hospital, Capital Medical University, Beijing 100070, China

Diffuse glioma is the most common primary malignant brain tumor in adults. Currently, the prognosis of glioma remains dismal, and almost all patients with glioma experience recurrence even after comprehensive treatment including maximal surgical resection, radiotherapy, and/or chemotherapy^1^. Gliomas can be stratified according to their IDH mutation status. As we have previously reported^2,3^, the genetic characteristics, pathogenesis, and chemotherapy response are distinct between IDH-mutant and IDH-wildtype tumors. For instance, IDH-wildtype tumors are associated with poor prognosis and frequently have genomic alterations, including a gain of chromosome 7 and loss of chromosome 10. IDH-mutant tumors often show findings including CpG island hypermethylation and MET alterations. The median overall survival of the most aggressive type IDH-wildtype glioblastoma (GBM), which accounts for more than 60% of gliomas, is less than 20 months^1^, mainly because GBM’s distinctive anatomic location, biological characteristics, genetic alterations, and microenvironmental features often render it resistant to conventional treatments. Therefore, alternative therapeutic strategies are urgently needed to improve patient prognosis^4,5^. In recent years, many efforts have attempted to target the classical hallmarks of tumors that were first summarized by Hanahan and Weinberg in 2000^6^ and further extended in 2011^7^. However, heterogeneity within the tumor microenvironment (TME) poses a challenge to treatment efficacy, and the detailed mechanisms underlying this dynamic diversity during treatment remain elusive.

Notably, the advent of single-cell sequencing technology has enabled in-depth characterization of the cellular states of tumor cells and their TME (**Table 1**), and may fundamentally deepen understanding of the mechanisms underlying treatment resistance and rapid relapse. As recently discussed by Hanahan in *Cancer Discovery*^15^, tumor hallmarks now include 4 new dimensions, including phenotypic plasticity, senescent cells, and non-mutational epigenetic reprogramming. These features are associated with tumor heterogeneity in response to the changing TME, and have been partially validated in IDH-wildtype glioma by several large-scale single-cell studies. For instance, our previous study^4^ has classified GBMs into 3 subtypes and demonstrated that the mesenchymal and proneural subtypes tend to exhibit high accumulation in recurrent GBMs, thus suggesting that each subtype may endow glioma with a specific ability to contribute to treatment resistance. This finding was further extended by Neftel et al.^9^, through an approach integrating single-cell RNA-sequencing, single-cell lineage tracing, and other methods. The authors found that tumor cells’ plasticity enables dynamic switching to 4 cellular states in response to genetic, microenvironmental and therapeutic stimuli. Moreover, Ravi et al.^16^ have shown that GBM has segregated niches characterized by distinct immunological and metabolic stress factors, thereby indicating the effects of the microenvironment on the development of tumor cells. Importantly, hypoxia induces defined transcriptional and genomic responses, including copy number alterations, thus suggesting that the TME affects not only transcript plasticity but also genetic alterations in gliomas.

**Table 1:** Current studies on tumor plasticity and the TME based on single-cell sequencing technologies

Highlights	Technology	Reference
GBM heterogeneity deconvolution	Single-cell RNA sequencing	Patel et al.^8^
GBM subtyping	Single-cell RNA sequencing	Wang et al.^4^
GBM cellular states and plasticity	Single-cell RNA sequencing	Neftel et al.^9^
Epigenetic heterogeneity and cellular plasticity in GBM tumor cells	Single-cell DNA methylation sequencing, single-cell RNA sequencing	Johnson et al.^10^
Glioma epigenetic encoding, inheritance, and transition dynamics	Single-cell DNA methylation sequencing, single-cell RNA sequencing	Chaligne et al.^11^
Interactions between macrophages and tumors, promoting mesenchymal transition	Single-cell RNA sequencing	Hara et al.^12^
Functional heterogeneity of glioma and glioma-associated immune cells	Single-cell RNA sequencing	Abdelfattah et al.^13^
Evolution of immune landscape during GBM progression	Single-cell RNA sequencing	Yeo et al.^14^

IDH-wildtype gliomas appear to have higher plasticity than IDH-mutant tumors. Johnson et al.^10^ have shown that tumor plasticity is accompanied by non-mutational epigenetic reprogramming, as confirmed through integrated analysis including single-cell DNA methylomes and single-cell transcriptomes. In both hypoxia and irradiation experiments, IDH-wildtype tumor cells have a tendency toward exhibiting perturbed DNA methylation, which is associated with cellular state shifts. Moreover, these findings are more likely to occur in IDH-wildtype tumors, which exhibit greater genomic instability than IDH-mutant tumors. A similar finding by Chaligne et al.^11^ has indicated that the heritability of malignant cell states shows key differences in hierarchal and plastic cell state architectures between IDH-mutant glioma and IDH-wildtype tumors, and IDH-wildtype tumors have higher cellular plasticity than IDH-mutant cells. Moreover, the Glioma Longitudinal AnalySiS (GLASS) Consortium has demonstrated that glioma progression is shaped by both genetic evolution and microenvironment interactions, *via* analysis of transcriptional profile alterations in more than 300 longitudinal cases^17^. Recurrent IDH-wildtype tumors show up-regulation of neuronal signaling programs or epithelial-mesenchymal transition pathways *via* interaction with myeloid cells, whereas recurrent IDH-mutant tumors often show a proliferative phenotype accompanied by CDKN2A deletion or hypermutation. Overall, tumor plasticity is an emerging hallmark of gliomas for maintaining the distribution of various cellular states, and is highly associated with genetic features and microenvironment interactions.

Among the cellular states, the senescent-like state is now receiving attention for its versatile roles in preventing apoptosis, inducing angiogenesis, promoting invasion, and suppressing immunity^15^. Senescent cells are induced by pressures such as hypoxia, but the fully viable tumor cell phenotype recovers after the TME changes, thus resulting in rapid disease progression. By activating the senescence-associated secretory phenotype (SASP), senescent cells release cytokines (e.g., CCL2), which recruit immune cells, such as macrophages, that infiltrate into tumor tissue^18^. Macrophages are a major component of the TME (30%–50%) and are widely involved in treatment resistance^4^ and immunosuppression^19^. Furthermore, macrophages have recently been reported by Hara et al.^12^ to secrete OSM, which in turn interacts with OSMR or LIFR in complex with GP130 on GBM cells, thus activating the STAT3 signaling pathway and inducing tumor cells to switch to a mesenchymal-like state—a phenotype associated with macrophage recruitment^4^. Therefore, a forward feedback loop of interactions between tumor cells and macrophages may sustainably promote tumor plasticity and immune cell enrichment (**Figure 1**).

**Figure 1:**
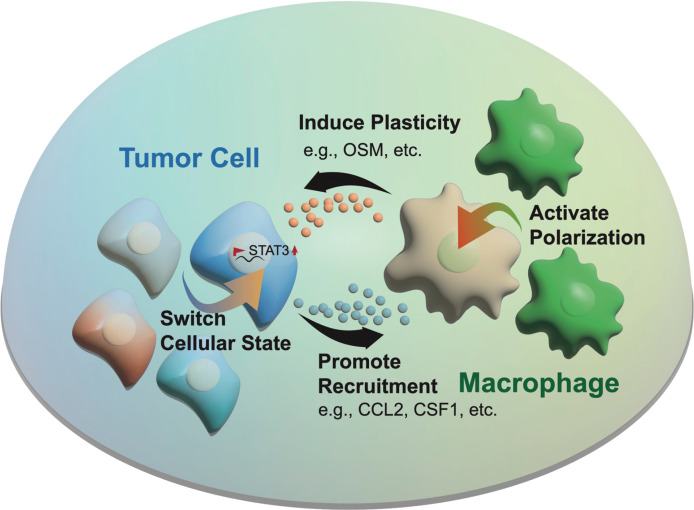
Forward feedback of the interplay between tumor cells and macrophages in promoting tumor progression. The loop includes 4 steps: (1) tumor cells promote macrophage recruitment through factors including CSF1 and CLL2; (2) macrophages activate polarization; (3) the polarized macrophages induce tumor plasticity by releasing factors including OSM; (4) and tumor cells switch cellular states (e.g., mesenchymal state).

## Perspectives

**Figure 2:**
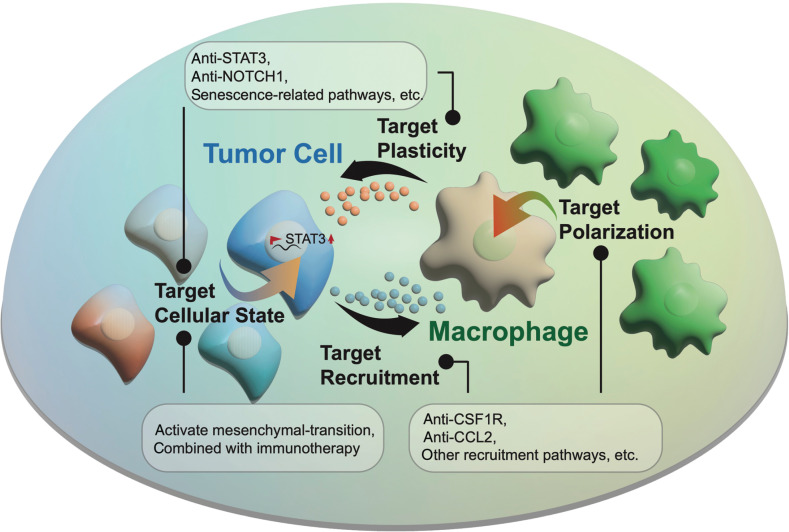
Potential treatment strategies: (1) blocking macrophage recruitment and polarization, such as with anti-CSF1R, anti-CCL2, and other potential recruitment/polarization pathways; (2) inhibiting tumor plasticity and intervening in tumor cellular state switching, such as with anti-STAT3 and anti-senescent cells. An alternative approach involves combining activating mesenchymal transition and immunotherapies.

Currently, a growing body of convincing published evidence^20,21^ indicates that the intense interplay between tumor cells and the TME has a pivotal role in maintaining angiogenesis, an immunosuppressive microenvironment, and tumor cell plasticity. Stromal cells such as cancer-associated fibroblasts remodel the surrounding extracellular matrix—a process increasing GBM invasion. However, the detailed molecule mechanism underlying this influence on tumors remains to be further investigated^22^. Endothelial cells are an essential component of the vasculature. The VEGF-driven signaling pathway is a well-known classical regulatory axis, which has an essential role in promoting angiogenesis and tumor growth. Substantial efforts have focused on targeting VEGF, but little progress has been made^23^. The development of immune therapy has spawned a series of new targeted therapies for reshaping the TME. For example, several studies^24,25^ have applied immune checkpoint inhibitors (e.g., anti-PD1) to reactivate immunity and kill tumor cells. Other efforts have focused on developing chimeric antigen receptor T cell immunotherapy or vaccines based on classical mutations (e.g., EGFRvIII) in tumor cells^26,27^. However, most of these studies have shown only limited improvements in prognosis. Given the versatile roles of macrophages in the TME, targeting macrophages has also been considered a promising strategy to prevent progression (**Figure 2**). Preclinical studies have used emactuzumab to target CSF-1R, a macrophage colony-stimulating factor receptor, thus successfully suppressing macrophage recruitment and the M2-like polarization phenotype in mice^28^. However, the recruitment pathways between tumor cells and macrophages are complex and diverse. Except for cytokines such as CCL2 attracts macrophages^18,29^, our previous study has indicated that NF1 deficiency may activate an alternative pathway inducing macrophage infiltration^4^. Therefore, systematic clarification of the mechanism of macrophage recruitment may provide more opportunities to identify potential strategies for improving prognosis. Other alternative strategies include direct inhibition of tumor plasticity. For example, regulators (e.g., STAT3 or NOTCH1)^12^ associated with the tumor state transition might be potential targets for treatment. Furthermore, systematic characterization of the molecular traits of senescent-like tumor cells with single-cell sequencing technology will also be essential to provide potential novel targets for remodeling the TME and preventing tumor relapse.
